# Two Sides of the Same Coin: Fluid Intelligence and Crystallized Intelligence as Cognitive Reserve Predictors of Social Cognition and Executive Functions Among Vulnerable Elderly People

**DOI:** 10.3389/fneur.2021.599378

**Published:** 2021-11-19

**Authors:** Natalia Salas, Josefina Escobar, David Huepe

**Affiliations:** ^1^Facultad de Educación, Psicología y Familia, Universidad Finis Terrae, Santiago, Chile; ^2^Center for Social and Cognitive Neuroscience, School of Psychology, Universidad Adolfo Ibáñez, Santiago, Chile

**Keywords:** cognitive reserve, fluid intelligence, crystallized intelligence, social cognition, executive functions

## Abstract

The concept of cognitive reserve –CR– postulates two forms that prevent cognitive impairment: neural reserve and neural compensation. Both have been primarily linked to the protective role played by genetic factors, educational level, occupation or socioeconomic status. Though it is true that it has been related to executive functions, so far very little attention has been paid to its predictive capacity with other variables more related to social cognition and psychosocial adaptation. Considering socially vulnerable contexts with reduced cultural capital and educational levels, the neural reserve function would be the most relevant and best predictor of aspects related to social cognition and executive functions. We suggest that variables such as fluid and crystallized intelligence influence social cognition and executive functions. This study included a sample of 27 participants over 60 years old from varied contexts of social vulnerability. The procedure included data collection using various cognitive measures. Results show that elderly people with high intelligence—mainly fluid intelligence—have better executive functions, emotional recognition and theory of mind. These results focus on cognitive reserve and its importance because they show that elderly people in vulnerable contexts who strengthen these aspects protect themselves against the deterioration of cognitive skills. This study is the first preliminary research to present a relationship between cognitive reserve and social cognition factors in elderly subjects. Fluid intelligence functions as a highly related factor to protect the performance of executive functions, along with other social-cognitive factors relevant to facilitating the conditions of social adaptation.

## Introduction

The concept of cognitive reserve –CR– (1, 2) involves two forms of protective actions. The first, known as the neural reserve, states that pre-existing brain networks that are more efficient or have a greater capacity may be less modifiable. On the other hand, it can operate as a neural compensation system where alternative networks can compensate for the disruption of pre-existing pathology networks. Thus, CR has mainly been linked to the protective role of education, occupation, or socio-economic status against cognitive impairment and very little attention has been paid to its moderating role, among other variables like social cognition, since it not only incorporates environmental aspects but also intrinsic body actions [concept differs "brain reserve capacity” - BRC - of (3)]. Epidemiological evidence suggests that individuals with higher fluid intelligence (FI), education, occupational level, or participation in leisure activities have a lower risk of developing Alzheimer's disease (AD) and other neurodegenerative diseases (2). According to the above, we wanted to know which of these components, considered part of the cognitive reserve, better predict social adaptation capabilities. So far, no studies are known to have tested this question. SC and EF refer to the quality of life in terms of social and relational activities, as well as the subjective well-being of an individual in a given context, which, in turn, is a crucial skill for proper development and adaptation in contexts of social vulnerability. This concept includes multiple dimensions such as social behavior, emotional regulation and the development of social habits (4, 5). Psychosocial functioning represents an ecological approach to everyday adaptation and cognitive contexts interrelating cognition and emotion (6). We suggest that variables such as fluid intelligence (FI) and crystallized intelligence (CrI), as part of CR, influence social cognition (SC), emotional recognition (ER), and executive functions (EF) in vulnerable contexts.

### Some Aspects on Elderly Chileans and Vulnerable Contexts

Chile has 2.8 million people 60 years of age and older [16.2% of the total Chilean population; (7)]. According to the Chilean National Health Survey 2009–2010 (8), prevalence of cognitive impairment in this group was 10.4% and it rises rapidly with advancing age (12.8% for people 70–79 years old and 20.9% for 80 years and over). Moreover, this prevalence is much higher among older adults living in contexts of social and economic vulnerability and with low educational levels; 39% had some degree of disability. From a socioeconomic standpoint, 44% of disabled people in Chile belong to low socioeconomic sectors. Older adults account for 44.3% of disabled people, with a high percentage having low levels of education [69.6% according to (9)]. The same trend was seen in the educational level, where prevalence of cognitive impairment was 5.6 times higher among less educated adults than among those with higher educational levels (8). Disability to perform in daily life among the elderly has the same distribution by socioeconomic and educational levels; which means that the poorest and least educated have a higher prevalence of disability to perform in everyday life (9). There is also a high prevalence of depression in Chile (17.5% according to the latest National Health Survey ENS Chile from 2009 to 2010), especially so among older adults (10) in vulnerable conditions (11). This is relevant, given the association between depression and development of dementia (12), for example. Among diseases associated with cognitive impairment, in Chile Alzheimer's disease is the leading cause of dementia in older adults. Its incidence increases with age (1–2% of the people aged 60 years, 3–5% of people 70, in 15–20% of people 80 years and one-third or half of those over 85). Its clinical course generally begins with failures in recent memory and ends with total dependence (13). Chilean authorities consider factors such as low education and low levels of culture (low-income patients from vulnerable and rural contexts) to be social determinants of this kind of disease (14). Social vulnerability is understood as a set of social characteristics that put a group of people who live in contexts of lower economic resources and at high risk of falling into poverty in a situation of structural, material and personal inequality (15, 16). Typically, these populations have low cultural and educational capital, limited access to goods and services, lower quality of social benefits (health, education, housing), higher levels of social deprivation, more impoverished social environments, and unsafe neighborhoods, effectively lowering the quality of life of their inhabitants compared to the general population (17, 18). For example, it is known that environments of lower social capital or poverty are more likely to be exposed to higher toxins and pollutants, crime and traffic and have fewer chances to participate in physical activities, less access to healthy food, greater likelihood of living in chaotic homes, more violence and lower parental sensitivity (19). Thus, exposure to multiple stressors, that is, various risk factors, has greater side-effects on cognitive abilities than exposure to a single risk (20, 21).

### Cognitive Reserve and Social Cognition as Associated Factors to Psychosocial Adaptation (PSA)

The term psychosocial adaptation (PSA) refers to quality of life in terms of social and relational activities. This concept includes multiple dimensions such as social behavior, emotional regulation, and the development of social habits (4, 5). PSA represents an ecological approach to everyday and cognitive-contextual adaptation, in which cognition and emotion are interrelated (6). Within PSA, social cognition (SC), fluid, and crystalized intelligence (FI; CrI) play a highly significant part, particularly in decision-making, emotional processing, and the way we relate to others (empathy and theory of mind). Research suggests that the prefrontal cortex plays a major role in such adaptability, given its involvement in the flexibility of behavior, executive functions (EF), FI and SC (22, 23). It should be noted that not all EF are related to FI (24–26). Similarly, SC tasks and FI (27, 28) or mental flexibility have been associated with this area (29, 30). Damage or alterations in the frontal lobe have direct implications on these functions, resulting mainly in maladaptive behaviors. SC includes the ability to make decisions, emotional processing, the ability to understand others' intentions and to develop in the social world (31). Studies in this line have shown that the social context exerts a profound influence on SC (32, 33). Meanwhile, FI has been defined as the ability to think logically and solve problems in new situations, regardless of the acquisition of knowledge. This reflects the ability to reason and think abstractly, in contrast to what is called crystallized intelligence (CrI) (34), which depends on cultural and academic learning. From a neuroanatomical viewpoint, FI has been associated with frontal lobe functions (27). Injuries in this area affect the performance of these cognitive abilities (28, 35) and studies that have measured FI with functional neuroimaging have shown activation of frontal areas (36, 37). FI has been linked as a protective factor for mental health, violent conditions and PSA (38). On the other hand, CrI is known as the ability to use skills, knowledge, and experience (39), relying on accessing information from long-term memory. As McGrew (40) establishes, it “is primarily a store of verbal or language-based declarative (knowing what) and procedural (knowing how) knowledge acquired through the investment of other abilities during formal and informal educational and general life experiences” (p. 5). According to this, CrI is indicated by a person's depth and breadth of general knowledge, vocabulary, and the ability to reason using words and numbers. As such it is also viewed as the product of educational and cultural experience in interaction with FI. One relevant element is that it changes with age (41, 42). For example, research on individuals over the age of 60 showed that many abilities indeed show average decline rates (43–45). Thus, given the description of SC, FI and CrI, this gains relevance as protective factors in aging, in addition to cognitive reserve (CR). In this sense, the CR can be defined as “differences in cognitive processes as a function of lifetime intellectual activities and other environmental factors that explain differential susceptibility to functional impairment in the presence of pathology or other neurological insult” [(46) p. 502]. CR acquires importance since it proposes two forms of protective actions (1, 2). Neural reserve refers to pre-existing brain networks that are more efficient or have a greater capacity but are less susceptible to change. It can also operate like an alternative neural network that compensates for the disruption of pre-existing pathology networks. Despite the fact that CR has mainly been linked to the protective role in everyday activities, as well in education, work, or socio-economic status against cognitive impairment (47), literature on SC shows that very little attention has been paid to its moderating role. It has even been suggested that there is no relationship (48, 49). This is interesting, considering that it incorporates environmental aspects as well as intrinsic body actions [concept differs “brain reserve capacity” (BRC) of (3)]. Epidemiological studies have shown that individuals with higher FI, educational and occupational levels or leisure-related activities have a lower risk of developing AD and other neurodegenerative diseases (2). In the same way, SC has been associated with positive outcomes in aging. On the one hand, factors such as positive life events and social support, have proven to influence and increase survival rates in patients with dementia compared to subjects living in poor conditions (50). Similarly, Fratiglioni et al. (51) have found that the construction of social networks with poor or limited support increased the risk of dementia by 60% and that there is a link between variables such as isolation, feelings of social isolation, educational level, among other lifestyles, that accelerate or delay the occurrence dementia (52, 53). These can be seen as protective factors, but with an active role (1) based on neural networks. On the other hand, evidence from epidemiological research suggests that higher FI, high educational and occupational levels (better jobs), being active in leisure activities or larger social networks, have a lower risk of developing, for example, Alzheimer's disease (AD) or dementia (54, 55). Therefore, we understand that people with better social adaptability, given their greater social cognition, will be more resistant to neurodegenerative diseases, either due to the existence of a more functional network or a larger brain capacity to involve alternative networks. There are known risks factors for neurodegenerative diseases such as genetic or medical conditions, other neurological pathologies, or brain injuries. Nevertheless, the role of social variables have been studied less formally and are less understood, especially considering only individual factors (56). One possible hypothesis about the relationship between CR and FI is that other protective social and cultural factors could modulate the adaptation process, benefiting from the social and cognitive conditions that people possess, such as social support and social networks, demographic aspects, educational level, among others. Although the concepts of CR, CrI and FI differ in important respects, they are complementary as opposed to competing. Accordingly, the study of social factors as predictors of the delay or acceleration of neurodegenerative diseases is essential and we sought to fill a gap in previous research by conducting a study to find the relationship between fluid intelligence (FI) and crystallized intelligence (CrI), and as complementary factor with cognitive reserve (CR), as well as their relationship to social cognition (SC), emotional recognition (ER) and executive function (EF) in older adults in socially vulnerable contexts. In addition, we want to go into greater depth and answer the question of which of these two types variables (FI or CrI) better predicts social adaptive capacity. We suggest that FI and CrI, are associated with aspects of SC, such as emotional recognition and theory of mind (ToM), in addition to EF; and could strength cognitive process that are crucial for CR. All these aspects could influence the processes of social adaptation and thus the possibility to the adjustment of the elderly in vulnerable contexts. CR is often estimated using proxy variables for lifetime exposures and cognitive activity: years of education, measures of crystallized intelligence, such as vocabulary or knowledge, literacy level, number of intellectually stimulating leisure activities, degree of occupational complexity, and socioeconomic status are all commonly used to create an estimate of CR (1). According to the above, FI and Crl are variables that we propose to use as a synthetic way to explain a broader construct of CR (since both variables are components of CR).

## Methods

### Participants

All twenty-seven participants in this study were in healthy conditions, over 60 years old (*M* = 66.44, *SD* = 6.59; 55.6% male) and recruited from contexts of social vulnerability. Given that they come from contexts of high social risk (poverty and insecurity), they are participants who are very difficult access. They come from families currently participating in a Chilean Social Security Program—CSPP—implemented by the Chilean Ministry of Social Development. All participants signed an informed consent, following the protocol of the Declaration of Helsinki. Participation was voluntary, protecting participants' anonymity. The following exclusion criteria were considered: individuals with visual and/or hearing impairment that prevents them from performing the various tasks and measurements in the study; and a psychiatric or neurological background representing an impediment to the evaluation of the protocol, assessed in an initial interview.

## Measures and Procedure

The procedure included direct telephone contact, with the help CSPP agencies, and data collection by members of the program, who had been previously trained in the measures taken. A house for neighborhood meetings (local area) was the setting for data collection and the interview considered a research assistant to complete the instruments. The study protocol included: Executive functions (EF), measured with INECO Frontal Screening –IFS- (57, 58), a brief tool that evaluates EF through different domains: programming tasks Motorboat; conflictual instructions, inhibitory verbal control, abstraction, back span of digits for working memory space, and Go / No Go testing. It is a very sensitive instrument and has been tested in patients with frontal and neuropsychiatric disorders and injuries. To measure Fluid and crystallized intelligence (FI and CrI), we used the Wechsler Adult Intelligence Scale III (WAIS-III) (59) and ran two subtests, progressive matrices and vocabulary. We obtained a total score for each subtest from each scale. The progressive matrices represent FI and from vocabulary test, we calculated CrI. Additionally, years of education also was used as a proxy for CrI (as a second variable) and Theory of Mind (ToM) was measured with Reading the Mind in the Eyes Test (60, 61). This battery evaluates ToM, through 28 pictures of faces of people where only the area around the eyes is visible;. Emotional recognition was tested with the Mini-Sea (62). This instrument is built around two subtests: (a) a facial emotion recognition test (from Ekman pictures; scored from 0 to 15) in which participants must identify which emotion is being expressed; and (b) a shortened version of the Faux Pas recognition test (63) to evaluate emotional recognition. Inhibitory verbal control was measured because it could be linked to emotional regulation and adaptation (64, 65). For that purpose, we used the Hayling Test (66), consisting in two parts: (a) concentrated attention, verbal initiation, processing speed and the strategy of a well-succeeded search for automated words; and (b) EF components, such as verbal inhibition and planning (the individual must inhibit the content of the sentence).

### Statistical Analysis

We obtained descriptive statistics (central trend and dispersion measurements) and subsequently calculated Pearson's correlation coefficients to evaluate the independency between variables. A Kolgomorov-Smirnov Test was conducted to test for normal distribution. We worked with p level of <0.05 (two-tailed) to confirm bivariate correlations. In addition, we quantified the evidence to support the alternative hypothesis by computing a Bayes factor for the specific effect of crystallized and fluid intelligence on: (a) EF score; (b) total emotional recognition score; (c) ToM; and (d) inhibitory verbal control. Bayes factor analysis for each prediction will yield very strong evidence to support the alternative expected effect on main interest variables when BF10 > 30 and ≤ 100; strong when BF10 > 10 and ≤ 30; and moderate evidence if it is between BF10 > 3 and BF ≤ 10 [assuming a uniform distribution of priors; see (67, 68)].

## Results

**Figure 1:**
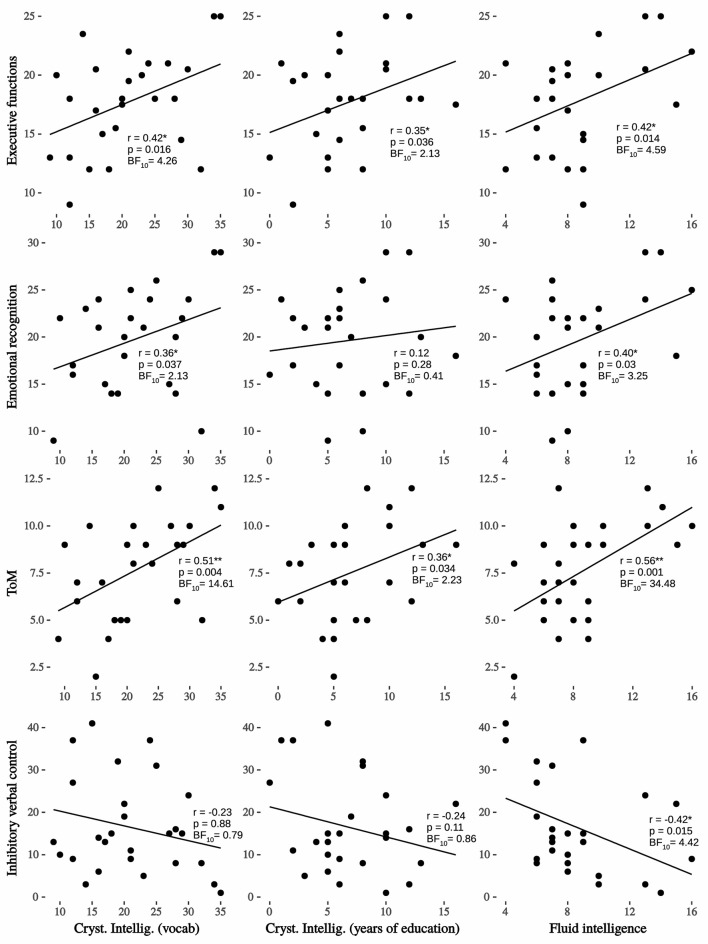
Regression lineal, Pearson's r and Bayesian factor between crystallized and fluid intelligence on interest variables. Very strong evidence between fluid intelligence and ToM; moderate evidence with executive functions, inhibitory verbal control and emotional recognition; strong evidence between crystallized intelligence (vocabulary) and ToM; moderate evidence between crystallized intelligence (vocabulary) and executive functions; ^*^p < 0.05; ^**^p < 0.01.

Descriptive statistics are shown in Table 1, where one can see that almost all the scores for each test are above the median point of the maximum obtained for its scale (with de exception of inhibitory verbal control and CrI). In Figure 1, Cognitive reserve (CR) variables –FI and CrI—show associations with social and cognitive variables. The most important effect found was between FI and ToM (*r* = 0.56, *p* < 0.01; BF_10_ = 34.48, very strong evidence) and next with executive functions (*r* = 0.42, *p* < 0.05; BF_10_ = 4.59, moderate evidence), inhibitory verbal control (*r* = 0.42, *p* < 0.05; BF_10_ = 4.42, moderate evidence) and emotional recognition (*r* = 0.40, *p* < 0.05; BF_10_ = 3.25, moderate evidence). On the other hand, and taking the Bayes factor into account, only crystallized intelligence (vocabulary) was significantly associated with ToM (*r* = 0.51, *p* < 0.01; BF_10_ = 14.61, strong evidence) and executive functions (*r* = 0.42, *p* < 0.05; BF_10_ = 4.26, moderate evidence). On the other hand, it is known that fluid intelligence and age are variables typically that have been inversely associated (15, 41, 69, 70). Before age 50 ability such as processing speed, memory, and reasoning begins to decline (71). To control the effect of the age, we calculate partial correlation for each variable of the interest, adjusting for age. Outcomes show that all correlations remained statistically significant (one-tailed): FI – FE, *r* = 0.41, *p* = 0.019; FI – ToM, *r* = 0.56, *p* = 0.001; FI – Emotional recognition, *r* = 0.35, *p* = 0.043; and inhibitory verbal control, *r* = −0.40, *p* = 0.021.

**Table 1:** Mean, standard deviations, median and minimum-maximum for each interest variable.

	**Executive function**	**Emotional recog**.	**ToM**	**Inhibitory verbal control**	**Crystallized intell (years education)**	**Crystallized intell (vocab.)**	**Fluid intelli**.
Mean	17.72	19.65	7.593	16.44	6.852	21.00	8.593
Std. deviation	4.163	5.321	2.606	11.28	3.870	7.514	3.153
Median	18.00	20.50	8.00	14.00	6.00	20.00	8.00
Minimum	9.00	9.00	2.00	1.00	0.00	9.00	4.00
Maximum	25.00	29.00	12.00	41.00	16.00	35.00	16.00

## Conclusion and Future Directions

This study shows that elderly individuals living in vulnerable contexts in Chile and with high intelligence -mainly fluid intelligence- have better executive functions, emotional recognition and theory of mind (ToM). This is the first preliminary research to present a relationship between cognitive reserve (CR), social cognition (SC) and executive function (EF) in elderly subjects who live in vulnerable contexts. As seen in the results, FI functions as a factor highly related to the improvement of EF shown by the subjects measured, along with other SC factors relevant in the probability of enhanced psychosocial adaptation (PSA). These results focus on cognitive reserve (IF and CrI connected to SC) and their possible protective effects in vulnerable contexts. We can say that older people in vulnerable contexts who strengthen these aspects (through CR) protect the deterioration of some cognitive and social abilities (72–74). Social interactions that promote the resolution of everyday situations (that is, FI) allow the brain to continue with the demands it has become accustomed to since birth. The exercise of cognitive aspects and the use of cognitive functions such as attention, memory and planning to solve day-to-day dilemmas function as protective factors in vulnerable contexts. This could also be because these contexts present multiple challenges that are approached from social and cognitive abilities and which generate greater social adaptation. Furthermore, social contact skills are essential for collaborative work and the joint construction of learning (75). Elderly people who adapt and continue learning use CR as a key element. This study invites us to review social and health policies regarding the promotion of programs for the elderly that favor autonomy and continuity in decision-making by the elderly. Activities that favor the continuity of establishing relationships with others, which allow elderly people to face the resolution of daily dilemmas, seem to be key and should be informed to those health professionals who attend to them.

Further studies that relate cultural and social factors associated with neurodegenerative diseases are needed, but not just as mere descriptors. On the other hand, SC should be increasingly considered in research on neurodegenerative diseases, not only as a potential early marker, but also as a key factor to understanding how it can moderate and slow the disease manifests itself on a day-to-day basis. Fluid intelligence must be revealed in its protective role, especially from its educational potential, in the sense that learning instances in the elderly should be highly promoted. The same way, SC and EF appear to be relevant factors to improving adaptive capacities, particularly in vulnerable contexts. There is a need to methodologically bridge the study of PSA skills between external individual characteristics (social and cultural factors) and internal (genetic or hereditary predisposition) based on the concept of “CR,” upon an active (neural networks) point of view.

Regarding the limitations of the present study, we can mention the small sample and the need for additional measurements to evaluate social adaptation. However, given the difficulty of access, and despite the small number of participants, it was still possible to robustly demonstrate the expected effects. In favor of this argument, other research types have been published with sample sizes similar to the present study (76–78). On the other hand, social adaptation was also considered through the protocol study that considered inclusion criteria associated with a population normally adapted to daily life.

In conclusion, this study has researched the positive effectiveness that CR has on SC and EF from an ecological perspective. Our results show that FI is the most relevant variable for predicting how elderly people adapt and function in their social environments.

## Data Availability Statement

The raw data supporting the conclusions of this article will be made available by the authors, without undue reservation.

## Ethics Statement

The studies involving human participants were reviewed and approved by Universidad Adolfo Ibáñez, Chile. The patients/participants provided their written informed consent to participate in this study.

## Author Contributions

NS and DH participated in the development of the study, in data collection and analysis, in writing and editing the manuscript, and in giving final approval. JE worked on editing the language and made important contributions to the manuscript and made final comments. All authors contributed to the article and approved the submitted version.

## Funding

This work was supported by grants from National Scientific and Technological Research Commission (ANID/FONDECYT Regular No. 1201486 to DH and ANID/FONDECYT Iniciación No. 11190565 to JE). The funders had no role in the decision to publish, or preparation of the manuscript.

## Conflict of Interest

The authors declare that the research was conducted in the absence of any commercial or financial relationships that could be construed as a potential conflict of interest.

## Publisher's Note

All claims expressed in this article are solely those of the authors and do not necessarily represent those of their affiliated organizations, or those of the publisher, the editors and the reviewers. Any product that may be evaluated in this article, or claim that may be made by its manufacturer, is not guaranteed or endorsed by the publisher.

## References

[1] Stern Y. What is cognitive reserve? Theory and research application of the reserve concept. J Int Neuropsychol Soc. (2002) 8:448–60. 10.1017/S1355617702813248 11939702

[2] Stern Y. Cognitive reserve and Alzheimer disease. Alzheimer Dis Assoc Dis. (2006) 20:112–7. 10.1097/01.wad.0000213815.20177.19 16772747

[3] Satz P. Brain reserve capacity on symptom onset after brain injury: a formulation and review of evidence for threshold theory. Neuropsychology. (1993) 7:273. 10.1037/0894-4105.7.3.273

[4] Bishop AJ Marteau TM Hall S Kitchener H Hajek P. Increasing women's intentions to stop smoking following an abnormal cervical smear test result. Prev Med. (2005) 41:179–85. 10.1016/j.ypmed.2004.09.046 15917009

[5] Cox KS Wilt J Olson B McAdams DP. Generativity, the Big Five, and psychosocial adaptation in midlife adults. J Person. (2010) 78:1185–208. 10.1111/j.1467-6494.2010.00647.x 20545818

[6] Wilson BA. Neuropsychological rehabilitation. Annu Rev Clin Psychol. (2008) 4:141–62. 10.1146/annurev.clinpsy.4.022007.141212 17716045

[7] Instituto Nacional de Estadistica I. Censo de Población y Vivienda. (2017). Available online at: https://redatam-ine.ine.cl/redbin/RpWebEngine.exe/Portal?BASE=CENSO_2002&lang=esp (accessed October 6, 2021)

[8] Ministerio de Salud de Chile. Encuesta Nacional de Salud ENS Chile 2009-2010. (2010). Available online at: http://web.minsal.cl/portal/url/item/bcb03d7bc28b64dfe040010165012d23.pdf (accessed October 6, 2021).

[9] Fondo Nacional de la discapacidad. Primer Estuido Nacional de la Discapacidad en Chile. Ministerio de Desarrollo Social y Familia, Gobierno de Chile (2005). Available online at: https://www.senadis.gob.cl/pag/136/1196/resultados_endisc_i (accessed October 6, 2021).

[10] von Mühlenbrock F Gómez R González M Rojas A Vargas L von Mühlenbrock C. Prevalencia de depresión en pacientes mayores de 60 años hospitalizados en el Servicio de medicina interna del hospital militar de santiago. Rev Chil Neuro Psiquiatría. (2011) 49:331–7. 10.4067/S0717-92272011000400004

[11] Ministerio de Salud de Chile. Plan Nacional De demencia 2017. (2017). Available online at: http://www.minsal.cl/wp-content/uploads/2017/11/PLAN-DE-DEMENCIA.pdf (accessed October 6, 2021).

[12] Snowden MB Atkins DC Steinman LE Bell JF Bryant LL Copeland C. Longitudinal association of dementia and depression. Am J Geriatric Psychiatry. (2015) 23:897–905. 10.1016/j.jagp.2014.09.002 25441056 PMC4369182

[13] Donoso A. La enfermedad de Alzheimer. Rev Chil Neuro Psiquiatría. (2003) 41:13–22. 10.4067/S0717-92272003041200003

[14] Servicio Nacional del Adulto Mayor. Estudio Nacional de la Dependencia en las Personas Mayores. (2010). Available online at: http://www.senama.cl/filesapp/EstudioNacionalde%0ADependenciaenlasPersonasMayores.pdf (accessed October 6, 2021).

[15] Chaudhuri S. Assessing Vulnerability to Poverty: Concepts, Empirical Methods and Illustrative examples. New York, NY: Department of Economics; Columbia University (2003). Available online at: http://www.econdse.org/wp-content/uploads/2012/02/vulnerability-assessment.pdf (accessed October 6, 2021).

[16] Henoch P. Vulnerabilidad Social. Más allá de la Pobreza. Serie Informe Soc, (2010) 128. Available online at: https://archivos.lyd.org/other/files_mf/SISO-128-Vulnerabilidad-social-mas-alla-de-la-pobreza-PHenoch-Agosto2010.pdf (accessed October 6, 2021).

[17] Neely-Prado A Navarrete G Huepe D. Socio-affective and cognitive predictors of social adaptation in vulnerable contexts. PLoS ONE. (2019) 14:1–23. 10.1371/journal.pone.0218236 31199834 PMC6568406

[18] Ministerio de Desarrollo Social. Informe de Desarrollo Social 2015. Gobierno de Chile (2015). Available online at: http://www.desarrollosocialyfamilia.gob.cl/pdf/upload/IDS_INAL_FCM_3.pdf (accessed October 6, 2021).

[19] Evans GW Kim P. Childhood poverty and young adults' allostatic load: the mediating role of childhood cumulative risk exposure. Psychol Sci. (2012) 23:979–83. 10.1177/0956797612441218 22825357

[20] Evans GW Kim P. Multiple risk exposure as a potential explanatory mechanism for the socioeconomic status–health gradient. Ann N Y Acad Sci. (2010) 1186:174–89. 10.1111/j.1749-6632.2009.05336.x 20201873

[21] Evans GW Li D Whipple SS. Cumulative risk and child development. Psychol Bull. (2013) 139:1342–96. 10.1037/a0031808 23566018

[22] Van Horn JD Irimia A Torgerson CM Chambers MC Kikinis R Toga AW. Mapping connectivity damage in the case of phineas gage. PLoS ONE. (2012) 7:e37454. 10.1371/journal.pone.0037454 22616011 PMC3353935

[23] Waters-Wood SM Xiao L Denburg NL Hernandez M Bechara A. Failure to learn from repeated mistakes: persistent decision-making impairment as measured by the iowa gambling task in patients with ventromedial prefrontal cortex lesions. J Int Neuropsychol Soc. (2012) 18:927–30. 10.1017/S135561771200063X 22643119

[24] Ardila A. Is intelligence equivalent to executive functions. Psicothema. (2018) 30:159–164. 29694315 10.7334/psicothema2017.329

[25] Friedman NP Miyake A Corley RP Young SE DeFries JC Hewitt JK. Not all executive functions are related to intelligence. Psychological Science. (2006) 17:172–9. 16466426 10.1111/j.1467-9280.2006.01681.x

[26] van Aken L Kessels RPC Wingbermühle E van der Veld WM Egger JIM. Fluid intelligence and executive functioning more alike than different? Acta Neuropsychiatrica. (2016) 28:31–7. 26281913 10.1017/neu.2015.46

[27] Duncan J Burgess P Emslie H. Fluid intelligence after frontal lobe lesions. Neuropsychologia. (1995) 33:261–8. 10.1016/0028-3932(94)00124-8 7791994

[28] Roca M Parr A Thompson R Woolgar A Torralva T Antoun N. Executive function and fluid intelligence after frontal lobe lesions. Brain. (2010) 133:234–47. 10.1093/brain/awp269 19903732 PMC2801324

[29] Larquet M Coricelli G Opolczynski G Thibaut F. Impaired decision making in schizophrenia and orbitofrontal cortex lesion patients. Schizop Res. (2010) 116:266–73. 10.1016/j.schres.2009.11.010 20022219

[30] Shamay-Tsoory SG Aharon-Peretz J Perry D. Two systems for empathy: a double dissociation between emotional and cognitive empathy in inferior frontal gyrus versus ventromedial prefrontal lesions. Brain. (2009) 132:617–27. 10.1093/brain/awn279 18971202

[31] Blakemore SJ Winston J Frith U. Social cognitive neuroscience: where are we heading? Trends Cogn Sci. (2004) 8:216–22. 10.1016/j.tics.2004.03.012 15120680

[32] Chung YS Barch DM. The effect of emotional context on facial emotion ratings in schizophrenia. Schizop Res. (2011) 131:235–41. 10.1016/j.schres.2011.05.028 21719258 PMC3872077

[33] Rankin KP Salazar A Gorno-Tempini ML Sollberger M Wilson SM Pavlic D. Detecting sarcasm from paralinguistic cues: anatomic and cognitive correlates in neurodegenerative disease. NeuroImage. (2009) 47:2005–15. 10.1016/j.neuroimage.2009.05.077 19501175 PMC2720152

[34] Cattell RB. The theory of fluid and crystallized general intelligence checked at the 5–6 year-old level. Brit J Educ Psychol. (1967) 37:209–24. 10.1111/j.2044-8279.1967.tb01930.x 6063107

[35] Woolgar A Parr A Cusack R Thompson R Nimmo-Smith I Torralva T. Fluid intelligence loss linked to restricted regions of damage within frontal and parietal cortex. Proc Natl Acad Sci USA. (2010) 107:14899–902. 10.1073/pnas.1007928107 20679241 PMC2930407

[36] Bishop S Fossella J Croucher C Duncan J. COMT val158met genotype affects recruitment of neural mechanisms supporting fluid intelligence. Cerebral Cortex. (2008) 18:2132–40. 10.1093/cercor/bhm240 18252743 PMC2517101

[37] Duncan J Seitz RJ Kolodny J Bor D Herzog H Ahmed A. A neural basis for general intelligence. Science. (2000) 289:457–60. 10.1126/science.289.5478.457 10903207

[38] Huepe D Roca M Salas N Canales_Johnson A Rivera_Rei AA Zamorano L. Fluid intelligence and psychosocial outcome: from logical problem solving to social adaptation. PLoS ONE. (2011) 6:e24858. 10.1371/journal.pone.0024858 21957464 PMC3177863

[39] Cattell RB. Theory of fluid and crystallized intelligence: a critical experiment. J Educ Psychol. (1963) 54:1. 10.1037/h0046743 6043849

[40] McGrew KS. CHC theory and the human cognitive abilities project: standing on the shoulders of the giants of psychometric intelligence research. Intelligence. (2009) 37:1–10. 10.1016/j.intell.2008.08.004

[41] Horn JL Cattell RB. Age differences in fluid and crystallized intelligence. Acta Psychol. (1967) 26:107–29. 10.1016/0001-6918(67)90011-X 6037305

[42] Belsky J. The Psychology of Aging: Theory, Research, and Interventions. Pacific Grove, CA: Brooks/Cole Pub. Co (1990).

[43] Finkel D Reynolds CA McArdle JJ Pedersen NL. Age changes in processing speed as a leading indicator of cognitive aging. Psychol Aging. (2007) 22:558. 10.1037/0882-7974.22.3.558 17874954

[44] Singer T Verhaeghen P Ghisletta P Lindenberger U Baltes PB. The fate of cognition in very old age: six-year longitudinal findings in the Berlin aging study (BASE). Psychol Aging. (2003) 18:318. 10.1037/0882-7974.18.2.318 12825779

[45] Whalley LJ Deary IJ Appleton CL Starr JM. Cognitive reserve and the neurobiology of cognitive aging. Ageing Res Rev. (2004) 3:369–82. 10.1016/j.arr.2004.05.001 15541707

[46] Barulli D Stern Y. Efficiency, capacity, compensation, maintenance, plasticity: emerging concepts in cognitive reserve. Trends Cogn Sci. (2013) 17:502–9. 10.1016/j.tics.2013.08.012 24018144 PMC3840716

[47] Stern Y. Cognitive reserve. Neuropsychologia. (2009) 47:2015–28. 10.1016/j.neuropsychologia.2009.03.004 19467352 PMC2739591

[48] Lavrencic LM Churches OF Keage HAD. Cognitive reserve is not associated with improved performance in all cognitive domains. Appl Neuropsychol Adult. (2018) 25:473–85. 10.1080/23279095.2017.1329146 28594578

[49] Lavrencic LM Kurylowicz L Valenzuela MJ Churches OF Keage HAD. Social cognition is not associated with cognitive reserve in older adults. Aging Neuropsychol Cogn. (2016) 23:61–77. 10.1080/13825585.2015.1048773 25989367

[50] Orrell M Butler R Bebbington P. Social factors and the outcome of dementia. Int J Geriatric Psychiatry. (2000) 15:515–20. 10.1002/1099-1166(200006)15:6<515::AID-GPS147>3.0.CO;2-U 10861917

[51] Fratiglioni L Hui-Xin W Ericsson K Maytan M Winblad B. Influence of social network on occurrence of dementia: a community-based longitudinal study. Lancet. (2000) 355:1315. 10.1016/S0140-6736(00)02113-9 10776744

[52] Eshkoor SA Hamid TA Nudin SSH Mun CY. The effects of social support, substance abuse and health care supports on life satisfaction in dementia. Soc Indic Res. (2014) 116:535–44. 10.1007/s11205-013-0304-0

[53] Holwerda TJ Deeg DJH Beekman ATF van Tilburg TG Stek ML Jonker C. Feelings of loneliness, but not social isolation, predict dementia onset: results from the Amsterdam study of the elderly (AMSTEL). J Neurol Neurosurg Psychiatry. (2014) 85:135–42. 10.1136/jnnp-2012-302755 23232034

[54] Bennett DA Schneider JA Tang Y Arnold SE Wilson RS. The effect of social networks on the relation between Alzheimer's disease pathology and level of cognitive function in old people: a longitudinal cohort study. Lancet Neurol. (2006) 5:406–12. 10.1016/S1474-4422(06)70417-3 16632311

[55] Crooks VC Lubben J Petitti DB Little D Chiu V. Social network, cognitive function, and dementia incidence among elderly women. Am J Public Health. (2008) 98:1221–7. 10.2105/AJPH.2007.115923 18511731 PMC2424087

[56] Andrew MK Rockwood K. Social vulnerability predicts cognitive decline in a prospective cohort of older Canadians. Alzheimer Dementia. (2010) 6:319–25. 10.1016/j.jalz.2009.11.001 20630414

[57] Ihnen J Antivilo A Muñoz-Neira C Slachevsky A. Chilean version of the INECO frontal screening (IFS-Ch): psychometric properties and diagnostic accuracy. Dementia Neuropsychol. (2013) 7:40–7. 10.1590/S1980-57642013DN70100007 29213818 PMC5619543

[58] Torralva T Roca M Gleichgerrcht E Lopez P Manes F. INECO frontal screening (IFS): a brief, sensitive, and specific tool to assess executive functions in dementia–CORRECTED VERSION. J Int Neuropsychol Soc. (2009) 15:777–86. 10.1017/S1355617709990415 19635178

[59] Wechsler D. WAIS-III: Wechsler Adult Intelligence Scale. 3rd editors. San Antonio, TX: Psychological Corporation (Spanish adaptation: WAIS-III: Escala Wechsler para adultos. Madrid: TEA 1998) (1997).

[60] Baron-Cohen S Wheelwright S Hill J Raste Y Plumb I. The “Reading the Mind in the Eyes” test revised version: a study with normal adults, and adults with asperger syndrome or high-functioning autism. J Child Psychol Psychiatry Allied Disc. (2001) 42:241–51. 10.1111/1469-7610.00715 11280420

[61] Brüne M Brüne-Cohrs U. Theory of mind—evolution, ontogeny, brain mechanisms and psychopathology. Neurosci Biobehav Rev. (2006) 30:437–55. 10.1016/j.neubiorev.2005.08.001 16239031

[62] Bertoux M Delavest M de Souza LC Funkiewiez A Lépine J.-P.. Social cognition and emotional assessment differentiates frontotemporal dementia from depression. J Neurol Neurosurg Psychiatry. (2012) 83:411–6. 10.1136/jnnp-2011-301849 22291219

[63] Stone VE Baron-Cohen S Knight RT. Frontal lobe contributions to theory of mind. J Cogn Neurosci. (1998) 10:640–56. 10.1162/089892998562942 9802997

[64] Carlson SM Wang TS. Inhibitory control and emotion regulation in preschool children. Cogn Dev. (2007) 22:489–510. 10.1016/j.cogdev.2007.08.002

[65] Rydell A-M Berlin L Bohlin G. Emotionality, emotion regulation, and adaptation among 5-to 8-year-old children. Emotion. (2003) 3:30. 10.1037/1528-3542.3.1.30 12899315

[66] Burgess PW Shallice T. The Hayling and Brixton Tests. St. Edmonds: Thames Valley Test Company Bury (1997).

[67] Albert J. Bayesian Computation With R. New York, NY: Springer Science & Business Media (2009).

[68] Ortega A Navarrete G. Bayesian hypothesis testing: an alternative to null hypothesis significance testing (NHST) in psychology and social sciences. Bayes Inference. (2017) 12:235–53. 10.5772/intechopen.70230

[69] Salthouse TA. Localizing age-related individual differences in a hierarchical structure. Intelligence. (2004) 32:541–61. 10.1016/j.intell.2004.07.003 24357886 PMC3866028

[70] Schubert AL Hagemann D Löffler C Frischkorn GT. Disentangling the effects of processing speed on the association between age differences and fluid intelligence. J Intellig. (2020) 8:1–20. 10.3390/jintelligence8010001 31881681 PMC7151009

[71] Bugg JM Zook NA DeLosh EL Davalos DB Davis HP. Age differences in fluid intelligence: contributions of general slowing and frontal decline. Brain Cogn. (2006) 62:9–16. 10.1016/j.bandc.2006.02.006 16603300

[72] Arcara G Mondini S Bisso A Palmer K Meneghello F Semenza C. The relationship between cognitive reserve and math abilities. Front Aging Neurosci. (2017) 9:429. 10.3389/fnagi.2017.00429 29311910 PMC5744435

[73] Evans IEM Llewellyn DJ Matthews FE Woods RT Brayne C Clare L. Social isolation, cognitive reserve, and cognition in healthy older people. PLoS ONE. (2018) 13:e0201008. 10.1371/journal.pone.0201008 30118489 PMC6097646

[74] Vance DE Roberson AJ McGuinness TM Fazeli PL. How neuroplasticity and cognitive reserve protect cognitive functioning. J Psycho Nurs Mental Health Serv. (2010) 48:23–30. 10.3928/02793695-20100302-01 20349891

[75] Singer T Seymour B O'doherty J Kaube H Dolan RJ Frith CD. Empathy for pain involves the affective but not sensory components of pain. Science. (2004) 303:1157–62. 10.1126/science.1093535 14976305

[76] Ciccarelli N Monaco MR Lo Fusco D Vetrano DL Zuccalà G. The role of cognitive reserve in cognitive aging: what we can learn from Parkinson's disease. Aging Clin Exp Res. (2018) 30:877–80. 10.1007/s40520-017-0838-0 29019160

[77] Héron M Le Faou A-L Ibanez G Métadieu B Melchior M. Smoking cessation using preference-based tools: a mixed method pilot study of a novel intervention among smokers with low socioeconomic position. Addic Sci Clin Practice. (2021) 16:1–10. 10.1186/s13722-021-00254-6 34193288 PMC8243481

[78] Tziakouri A Tsangari H Michaelides C. Assessment of the effect of erenumab on efficacy and quality-of-life parameters in a cohort of migraine patients with treatment failure in cyprus. Front Neurol. (2021) 12:687697. 10.3389/fneur.2021.687697 34393974 PMC8358110

